# Microbiota-Based Therapies for Recurrent Clostridium difficile Infection: A Systematic Review of Their Efficacy and Safety

**DOI:** 10.7759/cureus.90737

**Published:** 2025-08-22

**Authors:** Sasika Weerakoon, Sravanthi Avula, Bethel T Mandefro, Sri Vidya Sundara, Xinyu Lu, Hamide Busmail, Iana A Malasevskaia

**Affiliations:** 1 Internal Medicine, California Institute of Behavioral Neurosciences & Psychology, Fairfield, USA; 2 Pediatrics, NRI Medical College, Guntur, IND; 3 Medicine, California Institute of Behavioral Neurosciences & Psychology, Fairfield, USA; 4 Pediatrics, California Institute of Behavioral Neurosciences & Psychology, Fairfield, USA; 5 ENT, Shanghai Pudong Hospital, Shanghai, CHN; 6 Hospital-Based Medicine, Harvard T.H. Chan School of Public Health, Boston, USA

**Keywords:** fecal microbiota transplantation (fmt), gut dysbiosis, live biotherapeutic products, microbiome-based therapies, recurrent clostridioides difficile infection (rcdi)

## Abstract

Recurrent *Clostridium difficile *infection (RCDI) remains a significant clinical challenge, with high recurrence rates following standard antibiotic therapy. Emerging evidence supports the role of fecal microbiota transplant (FMT) and standardized microbiome therapeutics (e.g., SER-109, RBX2660) in gut microbiota restoration and recurrence prevention. This systematic review evaluates the effectiveness and safety of these approaches in comparison to traditional therapies.

Following the Preferred Reporting Items for Systematic reviews and Meta-Analyses (PRISMA) 2020 guidelines, we searched the databases PubMed/MEDLINE, ScienceDirect, Cochrane Library, Europe PubMed Central (Europe PMC), ClinicalTrials.gov, Google Scholar, and Elicit AI for studies published between January 2015 and May 2025. Eligible studies included randomized controlled trials (RCTs), observational studies, and case series assessing FMT in adults with rCDI. The risk of bias was assessed using the Cochrane Risk of Bias 2.0 tool (RoB 2) for RCTs and the Newcastle-Ottawa Scale (NOS) for cohort studies.

Seven studies (six RCTs, one cohort; N=1,030 patients) were included. FMT demonstrated superior efficacy compared to antibiotics/placebo, with clinical cure rates ranging from 70% to 91% (versus 23% to 62%). Donor FMT outperformed autologous FMT (90.9% vs. 62.5%, p = 0.042) and standard therapies (71% resolution vs. 33% fidaxomicin/19% vancomycin, p < 0.01). Microbiota-based therapies (SER-109, RBX2660) demonstrated comparable efficacy (RRR up to 68%). Safety profiles were favorable, with predominantly mild gastrointestinal events and no increased risk detected for the specific outcomes measured over a five-year follow-up period. Heterogeneity existed in administration routes (colonoscopy/capsules) and donor material (fresh/frozen).

FMT and standardized microbiome therapies are highly effective for treating rCDI, demonstrating robust short-term efficacy and favorable long-term safety. Donor-derived interventions and pharmaceutical-grade products (SER-109, RBX2660) represent promising alternatives to traditional antibiotics, particularly in recurrent or refractory cases. Future research should aim to standardize protocols and include more high-risk populations.

## Introduction and background

*Clostridium difficile (C. difficile)* is a Gram-positive, anaerobic, spore-forming bacterium and a major cause of healthcare-associated infections. It is typically acquired in hospital or long-term care settings, particularly following antibiotic exposure, which disrupts the normal gut microbiota (a state termed dysbiosis) and leads to *C. difficile* overgrowth [1]. In the United States, *C. difficile* is the most frequently reported nosocomial pathogen. A study in 2011 revealed 453,000 new cases of *Clostridium difficile* infection (CDI) and 29,000 deaths associated with CDI and related complications [2]. Recent CDC data indicate an incidence rate of 116.1 cases per 100,000 persons in 2022, and CDI rates increased with age and were more prevalent in women than in men, as well as in White individuals compared to others [3]. From minor, self-limiting diarrhea to potentially fatal fulminant colitis, CDI symptoms can be linked to bowel perforation, toxic megacolon, pseudomembranous colitis (a severe inflammation of the colon characterized by yellowish plaques on the mucosa), sepsis, or multiorgan dysfunction syndrome [4]. Over the past two decades, the disease burden has increased significantly, with rising incidence, severity, and associated healthcare costs.

Recurrent CDI (RCDI) is associated with significant morbidity, reduced quality of life, and increased healthcare utilization. Traditional antibiotic therapy, while initially effective, further disrupts the gut microbiota, perpetuating a cycle of recurrence. Recent data indicate that 15-30% of primary CDI patients experience rCDI after discontinuation of antibiotic therapy, and patients with multiple relapses (60% will relapse again) [5,6]. Furthermore, the annual rCDI incidence in the US is estimated to be between 75,000 and 175,000 new cases. The pathophysiology of rCDI involves persistent dysbiosis-an imbalance in gut microbial communities, impaired colonization resistance, allowing *C. difficile* spores to reemerge and produce toxins A and B, which damage the intestinal lining and trigger inflammation [7].

Traditional treatments for rCDI include prolonged or tapered courses of vancomycin or fidaxomicin, but these further disrupt the microbiota and do not address the root cause of recurrence [8]. One new and potential therapy option for rCDI is fecal microbiota transplantation (FMT). FMT involves transferring processed stool from a healthy donor into the gastrointestinal tract of an affected recipient to normalize gut microbiota and reverse dysbiosis [9]. By reestablishing a healthy microbial ecosystem, FMT suppresses *C. difficile* overgrowth, promotes mucosal healing, and supports immune regulation. Numerous studies have demonstrated that FMT is an effective treatment for recurrent and refractory CDI, even in patients who are immunosuppressed or have significant comorbidities [9]. In addition to traditional FMT, standardized microbiota-based therapies such as SER-109 and RBX2660 have recently emerged. These FDA-approved, pharmaceutical-grade products contain purified microbial consortia or live biotherapeutic agents designed to restore gut microbiota and prevent recurrence following antibiotic treatment [10,11].

This systematic review aims to evaluate and synthesize the current evidence on the safety and efficacy of FMT in patients with rCDI. It compares primary and secondary outcomes between patients treated with FMT and those who received standard antibiotic therapy or a placebo without FMT. By synthesizing the existing evidence on the safety and efficacy of FMT, we seek to provide insights that can guide future research directions.

## Review

Methods

Study Design

This systematic review adhered to the Preferred Reporting Items for Systematic reviews and Meta-Analyses (PRISMA) 2020 guidelines for reporting systematic reviews and meta-analyses [12]. The safety and effectiveness of FMT in patients with rCDI were examined in this review.

The research question, framed within the relevant PICO (Population, Intervention, Comparison, Outcome) framework, was as follows: What are the safety and efficacy outcomes of FMT in patients with rCDI compared to those with recurrent infection who have not undergone FMT? The PICO components were as Follows: Population - patients aged 18 years and older with rCDI who had undergone FMT; Intervention - FMT, including both traditional FMT and standardized, FDA-approved microbiota-based therapies; Comparison - patients with rCDI who did not undergo FMT; and Outcome - safety and efficacy outcomes of FMT.

Eligibility Criteria

We identified studies that assessed the safety and efficacy of FMT in patients with rCDI. The inclusion criteria were (1) Individuals with rCDI, aged 18 years or older. (2) Original studies: randomized controlled trials (RCTs), controlled clinical trials (CCTs), and observational studies (case-control studies, cohort studies, cross-sectional studies, case series, and case reports). (3) Studies published in English. (4) Moderate- and high-quality studies with a low risk of bias. (5) Studies published between January 1, 2015, and May 30, 2025. The exclusion criteria were (1) individuals without rCDI and individuals below 18 years old. (2) Studies involving animals and studies not matched with the PICO elements. (3) Non-original studies: posters, reviews, abstracts, editorials, and opinions. (4) Ongoing studies and studies finished without results. (5) Studies with a high risk of bias. (6) Studies published before January 1, 2015.

Search Strategy

A systematic search was conducted from May 15 to May 30, 2025. The following electronic databases were utilized: PubMed/MEDLINE, ScienceDirect, Cochrane Library, Europe PubMed Central (Europe PMC), and ClinicalTrials.gov. Additionally, a supplemental search was conducted on June 10, 2025, using Google Scholar and the Elicit AI Research Assistant website (https://elicit.com/ [13]).

The search strategy utilized specific keywords corresponding to the PICO elements. For fecal microbiota transplantation, keywords included “fecal microbiota transplant,” “fecal transplantation,” “stool transplant,” “stool bacteriotherapy,” and “donor feces transplant.” Recurrent infection was addressed with “disease recurrence,” “reinfection,” “repeat infection,” and “repeated episodes.” The search targeted *C. difficile* infection using terms such as “CDI,” “*C. diff*,” and “*Clostridium difficile*-associated diarrhea.” Safety and efficacy were addressed through keywords like “treatment outcome” and “effectiveness.” 

The search strategy also incorporated relevant Medical Subject Headings (MeSH terms) where applicable. Table 1 provides a detailed breakdown of the search strategy used for each database.

**Table 1:** Search strategy A thorough explanation of each database's search methodology CDI: *Clostridium difficile* infection; Europe PMC: Europe PubMed Central; FMT: fecal microbiota transplant; MeSH: Medical Subject Headings

Search strategy	Database	Filters	No. of studies	Date
(“Fecal microbiota transplant” OR “fecal transplantation” OR “stool transplant” OR “stool bacteriotherapy” OR “donor feces transplant” OR “FMI” OR “microbiota transfer” OR “fecal microbiota transplantation/adverse effects” [Mesh] OR “fecal microbiota transplantation/classification” [Mesh] OR “fecal microbiota transplantation/instrumentation” [Mesh] OR “fecal microbiota transplantation/methods” [Mesh] OR “fecal microbiota transplantation/standards” [Mesh] OR “fecal microbiota transplantation/trends” [Mesh] ) AND (“Recurrent infection” OR “Disease recurrence” OR “Reinfection” OR “Repeat infection” OR “Repeated episodes” OR "Reinfection/diagnosis"[Mesh] OR "Reinfection/microbiology"[Mesh] OR "Reinfection/therapy"[Mesh]) AND “Clostridium difficile infection” OR “CDI” OR “C. diff” OR “Clostridium difficile associated diarrhea” OR "Clostridium difficile/classification” [Mesh] OR “Clostridium difficile/drug effects” [Mesh] OR “Clostridium difficile/growth and development” [Mesh] OR “Clostridium difficile/pathogenicity” [Mesh]) AND (“Safety and efficacy” OR “Treatment outcome” OR “Effectiveness” OR "Treatment Outcome"[Mesh] OR "Treatment Expectations"[Mesh] OR “Patient Safety”)	PubMed/MEDLINE	Filters applied: in the last 10 years (2015-2025), full text, adaptive clinical trial, case reports, clinical study, clinical trial, controlled clinical trial, equivalence trial, evaluation study, multicenter study, observational study, pragmatic clinical trial, randomized controlled trial, English, humans	276	5/15/2025
ABSTRACT: ((("Fecal microbiota transplant" OR "fecal transplantation" OR "stool transplant" OR "stool bacteriotherapy" OR "donor feces transplant" OR "FMI" OR "microbiota transfer") AND ("Recurrent infection" OR "Disease recurrence" OR "Reinfection" OR "Repeat infection" OR "Repeated episodes") AND ("Clostridium difficile infection" OR "CDI" OR "C. diff" OR "Clostridium difficile associated diarrhea") AND ("Safety and efficacy" OR "Treatment outcome" OR "Effectiveness" OR "Patient Safety"))	Europe PMC	Last 10 years (2015-2025), research articles	71	05/16/2025
("Fecal Microbiota Transplantation" OR "Stool Transplantation") AND ("Reinfection" OR "Recurrent infection" OR "Disease recurrence" OR "Reinfection") AND ("Clostridioides difficile” OR “C. diff") AND ("Treatment Outcomes)	ScienceDirect Search 1	(2015-2025), research articles	3	5/16/2025
Search Hits #1 ("Fecal microbiota transplant" OR "fecal transplantation" OR "Stool transplant" OR "Stool bacteriotherapy" OR "Donor feces transplant" OR "FMI" OR "Microbiota Transfer") 531 #2 MeSH descriptor: [Fecal Microbiota Transplantation] explode all trees 241 #3 #1 OR #2 725 #4 ("Recurrent infection" OR "Disease recurrence" OR "Reinfection" OR "Repeat infection" OR "Repeated episodes") 4344 #5 MeSH descriptor: [Reinfection] explode all trees 37 #6 #4 OR #5 4344 #7 ("Clostridium difficile infection" OR "CDI" OR "C. diff" OR "Clostridium difficile associated diarrhea") 1794 #8 MeSH descriptor: [Clostridium Infections] explode all trees 850 #9 #7 OR #8 2320 #10 #3 AND #6 AND #9 12	Cochrane Library	(2015-2025), English trials	11	5/16/2025
Condition: ("Clostridium difficile infection" OR "CDI" OR "C. diff" OR "Clostridium difficile associated diarrhea") | Intervention: ("Fecal microbiota transplant" OR "fecal transplantation" OR "Stool transplant" OR "Stool bacteriotherapy" OR "Donor feces transplant" OR "FMI" OR "Microbiota Transfer")	ClinicalTrials.gov	Interventional, observational studies, and studies with results	22	5/16/2025
Research question: What are the safety and efficacy outcomes of fecal microbiota transplantation in patients with recurrent Clostridium difficile infection?	Google Scholar	(2015-2025)	20	6/10/2025
Research question: What are the safety and efficacy outcomes of fecal microbiota transplantation in patients with recurrent Clostridium difficile infection?	Elicit AI	(2015-2025) Randomized clinical trials, longitudinal studies	24	6/10/2025

*Screening and Quality Assessment* 

After the initial search, all identified records were imported into the Rayyan web application [14]. The screening process was conducted independently, and duplicates were removed. The collected records were then initially screened by evaluating their titles and abstracts. Disagreements were resolved through open discussion and a consensus-based approach. Full-text articles of potentially eligible studies were then retrieved and independently assessed against the eligibility criteria by the authors. Discussion and agreement were used to settle any outstanding disputes.

Data were extracted for each included study and independently verified for accuracy. Extracted elements included study design, population characteristics (such as age, sex, and disease severity), intervention details (including FMT method, dose, and frequency), comparator details, outcome measures (and their methods of measurement), and key findings (e.g., effect sizes, p-values). Any discrepancies in data extraction were resolved through discussion and consensus.

Risk of Bias Assessment

The risk of bias in included studies was assessed using the Cochrane Risk of Bias 2.0 tool (RoB 2) for RCTs [15] and the Newcastle-Ottawa Scale (NOS) for cohort studies [16]. Disagreements were resolved through open discussion and a consensus-based approach. Each domain of the chosen tool was assessed, and an overall risk of bias judgment was assigned for each study. While most studies were rated as low or moderate risk of bias using established tools (RoB 2 and NOS), several residual concerns merit attention. In some RCTs, blinding of participants and personnel was either incomplete or not reported, which may have introduced performance bias. Additionally, selective outcome reporting was suspected in a subset of studies where predefined endpoints were inconsistently presented. Funding sources were disclosed in most studies, with some industry-sponsored trials; although no overt bias was detected, this remains a potential source of influence. These factors underscore the need for cautious interpretation of efficacy and safety outcomes, particularly in studies rated as having "some concerns or moderate bias".

Data Synthesis and Analysis

A narrative synthesis was conducted to summarize the findings on the safety and efficacy of FMT for rCDI, given the observed heterogeneity across the included studies. This synthesis was structured around key efficacy outcomes, including primary and sustained cure rates, recurrence, and safety outcomes, such as the frequency and severity of adverse events.

Key characteristics of the included studies and quantitative outcome data (e.g., cure rates, adverse event frequencies) were presented in summary tables. The narrative text described the range and consistency of findings, explored potential reasons for heterogeneity, and highlighted robust or novel results. Charts and figures were used to visually supplement the data where appropriate. Because of the anticipated diversity and heterogeneity of the included studies, a formal meta-analysis was not conducted.

Results

**Figure 1:**
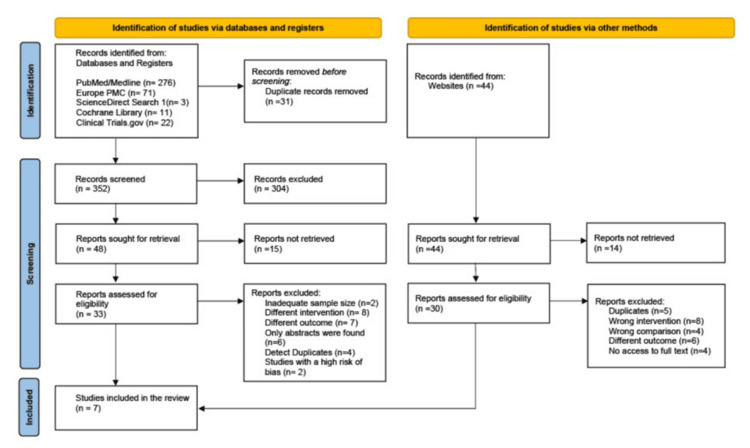
PRISMA flow diagram depicting the selection of studies Europe PMC: Europe PubMed Central; PRISMA: Preferred Reporting Items for Systematic Reviews and Meta-Analyses

Figure 1 presents the PRISMA flow diagram outlining the methodology and selection process. Out of 383 initially identified records, 31 duplicates were removed, leaving 352 records for screening. Following this initial review, a comprehensive evaluation of the full-text articles was conducted, resulting in the selection of four eligible studies. Additionally, three more studies were identified through Google Scholar and the Elicit AI Research Assistant (https://elicit.com/) [13].

Six of the included studies were RCTs; three of them were rated as low risk of bias across all five domains, reflecting well-conducted studies. Three of the studies were considered to have some concerns with deviation from the randomization process, deviation from the intended intervention, and bias in reporting results and outcomes. In summary, most studies demonstrate strong methodology, with a few representing moderate concerns that should be considered in interpreting their results. 

**Figure 2:**
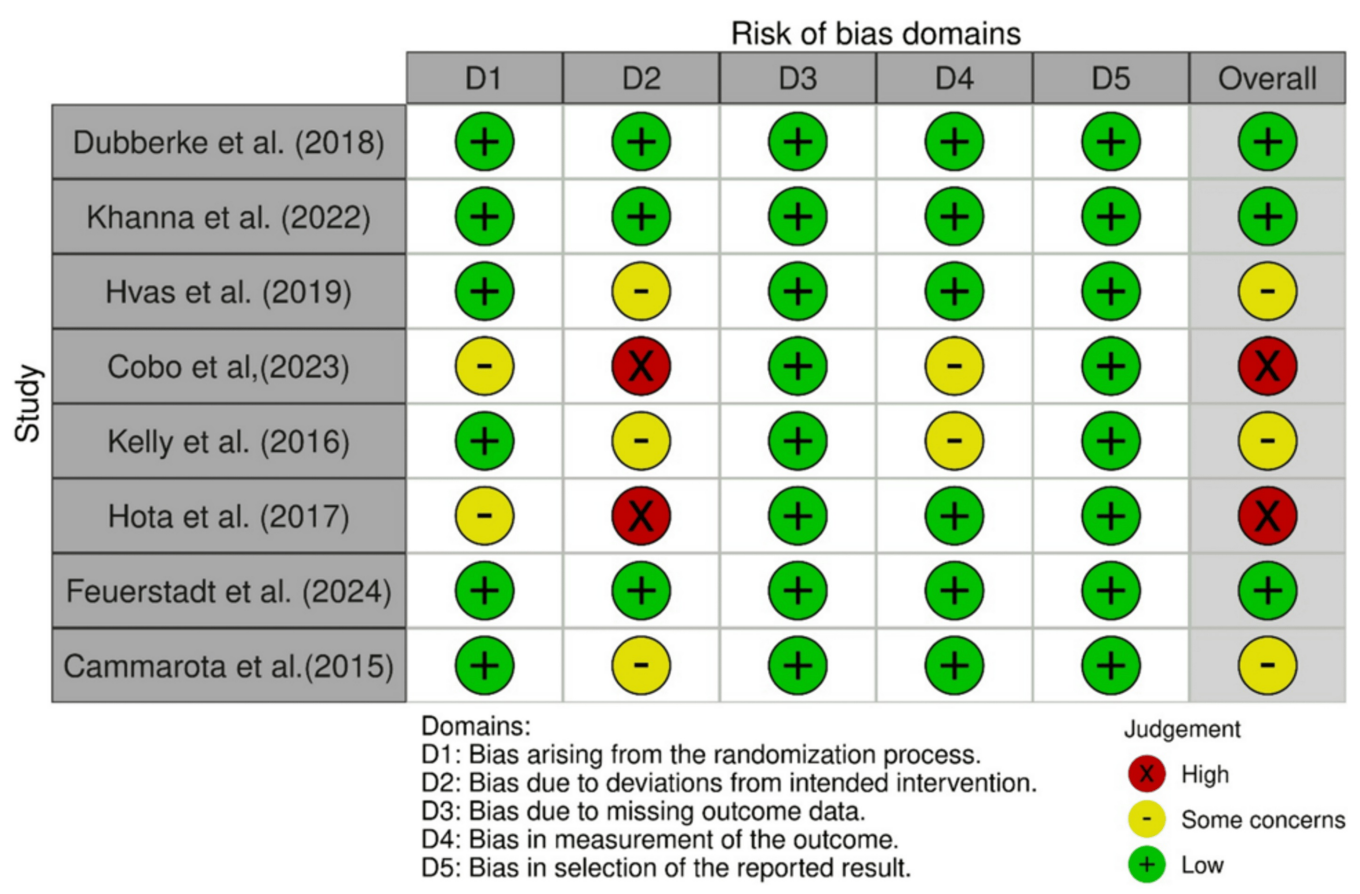
Quality appraisal of included RCTs using Cochrane Risk of Bias 2 Tool The figure illustrates the risk of bias evaluation for the randomized controlled trials (RCTs) using the Cochrane Risk of Bias 2.0 (RoB 2) tool Each domain is color-coded based on the level of risk: Green (+) indicates low risk of bias; Yellow (−) indicates some concerns; Red (×) indicates high risk of bias

A substantial risk of bias led to the exclusion of two RCTs from the systematic review. The intended intervention was not followed in these two investigations. The details of the quality assessment of RCTs are displayed in Figure 2 below.

Our single-cohort study was evaluated using NOS [16], resulting in a perfect score of 9 out of 9, which indicates a high level of study quality (Table 2).

**Table 2:** Quality appraisal of included cohort study using Newcastle Ottawa Scale For cohort studies, a maximum of four stars (*) could be awarded for selection, two for comparability, and three for outcome domains. The total score ranges from zero to nine. Passing score: 7/9

Study	Selection	Comparability	Outcome	Overall
Cold et al., 2022 [17]	****	**	***	9/9

Summary of Included Studies

A total of seven studies investigating the safety and effectiveness of FMT in the treatment of rCDI were included in this review. Six RCTs and one large retrospective cohort study make up the evidence base. With a combined total of more than 1,000 individuals across the principal analytic groups, the studies were primarily conducted in North America (the United States and Canada) and Europe (Denmark and Italy). The patient populations were consistently composed of adults with a history of rCDI, with a mean age typically in the mid-to-late 60s, reflecting the higher incidence of rCDI in older individuals.

Evaluating the effectiveness of FMT in avoiding additional CDI recurrences in comparison to a control group was the main goal of all six RCTs. For instance, Kelly et al. (2016) [18] and Cammarota et al. (2015) [5] compared traditional FMT against standard vancomycin therapy. More recent trials have focused on standardized, investigational microbiome therapeutics. Specifically, Khanna et al. (2022) [19] and Dubberke et al. (2018) [20] assessed the efficacy and safety of RBX2660, while Feuerstadt et al. (2024) [21] evaluated SER-109 against a placebo. The primary efficacy endpoint across the randomized controlled trials was treatment success, generally defined as the absence of rCDI recurrence during follow-up.

In contrast, the retrospective cohort study by Cold et al. (2022) [17] had a distinct primary aim focused on long-term safety. Together, these studies provide a comprehensive overview of both the short-term efficacy of various FMT-based therapies in preventing rCDI and their long-term safety profiles (Table 3).

**Table 3:** Characteristics of the included studies CDI: *Clostridioides difficile* infection; FMT: fecal microbiota transplantation; ITT: intention-to-treat; mITT: modified intention-to-treat; PP: per-protocol; RBT: rectal bacteriotherapy; rCDI: recurrent* Clostridioides difficile* infection; RCT: randomized controlled trial; RBX2660: an investigational microbiome-based therapeutic; SER-109: an investigational microbiome-based therapeutic consisting of purified Firmicutes spores

Study	Study design	Sample size	Country	Age	Aim of the study
Cold et al. (2022) [17]	Retrospective cohort study	280 patients (145 FMT, 135 RBT)	Denmark	Mean age for FMT group: 66.9 years. Mean age for RBT group: 66.7 years	FMT's long-term safety as a treatment for rCDI will be evaluated by contrasting it with RBT, a fixed bacterial mixture. The primary aim: to assess whether patients receiving FMT had a greater risk of dying, being admitted to hospital, or being diagnosed with a new onset of a certain specific predetermined disease compared to patients treated with RBT. The secondary aim: assessing if FMT treatment resulted in positive outcomes by lowering the requirement for treatment of specific disorders
Khanna et al. (2022) [19]	RCT	Total screened: 320; randomly assigned: 289; received blinded treatment: 267; ITT population: 267; mITT population: 262; PP population: 245	USA and Canada	19-93 years	The study aims to determine the effectiveness and safety of RBX2660 in preventing rCDI by assessing treatment success defined as the absence of *C. difficile* infection diarrhea within 8 weeks of study treatment
Dubberke et al. (2018) [20]	RCT	Total enrolled: 150; randomly assigned: 133; safety population: 128; ITT efficacy analysis: 127	United States and Canada	Group A: 24-89; Group B: 19-92; Group C: 18-88	The study's objective is to show that one or two doses of RBX2660 are safe and effective in preventing rCDI
Hvas et al. (2019) [22]	RCT	64	Denmark	21–92	This study evaluates the comparative efficacy of FMT, fidaxomicin, and vancomycin in managing rCDI
Feuerstadt et al. (2024) [21]	RCT	182	United States and Canada	18 years and older; mean age: 65.5 years	The study aimed to evaluate the efficacy of SER-109 in reducing the rCDI infection compared to a placebo, and to explore the concept of a two-pronged treatment approach combining antibiotics with microbiome therapy
Kelly et al. (2016) [18]	RCT	46 patients were randomly assigned; 43 completed the 8-week follow-up evaluation	United States	Age range between mid-30s and early 70s	To evaluate the safety and effectiveness of FMT in the treatment of rCDI
Cammrota et al. (2015) [5]	RCT	39	Italy	FMT group: 29–89 years; vancomycin group: 49–93 years	To study the effect of FMT via colonoscopy in patients with rCDI compared to the standard vancomycin regimen

Efficacy and Safety of Microbiota-Based Therapies

The efficacy of microbiota-based therapies in preventing rCDI was a primary outcome in five studies, with interventions demonstrating superiority over comparators. Specifically, Feuerstadt et al. found that the oral microbiome therapeutic SER-109 significantly reduced rCDI risk compared to placebo, with an overall relative risk (RR) of 0.32 (95% CI: 0.18-0.58; p < 0.001) and a sustained clinical response of 88% vs. 60% [21]. For the live biotherapeutic RBX2660, Khanna et al. reported a treatment success rate of 70.6% compared to 57.5% for placebo [14], while Dubberke et al. (2018), despite not meeting the primary endpoint (p = 0.152), found in a post-hoc analysis that at least one dose of RBX2660 was superior to placebo (p=0.047) [20]. Studies comparing FMT to standard care also showed significant benefits. Hvas et al. demonstrated a higher combined clinical resolution and negative toxin test rate for FMT (71%) vs. fidaxomicin (33%; p = 0.009) and vancomycin (19%; p = 0.001) [22]. Similarly, Cammarota et al. reported a 90% remission rate with FMT compared to 26% with a standard vancomycin regimen (p < 0.0001) [5]. Furthermore, donor FMT was found to have a significantly higher clinical cure rate (90.9%) than autologous FMT (62.5%; p = 0.042) in the study by Kelly et al. [18]. Regarding long-term outcomes, a five-year analysis by Cold et al. found no significant difference between FMT and rectal bacteriotherapy in survival, risk of hospital admission, or the onset of new diseases (Table 4) [17].

**Table 4:** Comparison of microbiota-based treatments for recurrent CDI in terms of long-term safety and effectiveness aHR: adjusted hazard ratio; AE: adverse event; CDI:* Clostridioides difficile* infection; CI: confidence interval; DM1: diabetes mellitus type 1; DM2: diabetes mellitus type 2; FMT: fecal microbiota transplantation; FMTv: fecal microbiota transplantation preceded by vancomycin; HR: hazard ratio; IBD: inflammatory bowel disease; MDRO: multidrug-resistant organism; NAAT: nucleic acid amplification test; OR: odds ratio; PCR: polymerase chain reaction; PMC: pseudomembranous colitis; RBT: rectal bacteriotherapy; RBX2660: live biotherapeutic product (microbiota-based drug); RR: relative risk; SAE: aerious adverse event; SER-109: oral microbiome therapeutic (purified Firmicutes spores)

Study	Intervention	Control/comparison	Reported primary outcomes	Reported secondary outcomes	Follow-up	Safety	Limitations
Cold et al. (2022) [17]	Various Danish FMT stool banks. Material: initially fresh, then frozen donor material after January 2018. Administration routes: capsules, enemas, upper or lower endoscopy. Donor type: multi-donor and single-donor capsules. Amount of stool used: varied from 50 to 200 g	RBT	Long-term safety of FMT compared to RBT by evaluating survival, hospital admission risk, onset of specific diseases (cancer, diabetes mellitus, hypertension, and inflammatory bowel disease). Survival: aHR = 1.03; 95% CI: 0.68-1.56; p = 0.89. Risk of hospital admission: aHR = 0.92; 95% CI: 0.72-1.18; p = 0.50. Cancer: HR = 1.82; 95% CI: 0.54-6.09; p = 0.33. DMT2: HR = 3.44; 95% CI: 0.38-31.02; p = 0.27. MDRO: HR = 1.05; 95% CI: 0.20-5.45; p = 0.96	Disappearance of disease/cessation of treatment: no significant difference between FMT and RBT groups (p = 0.58 for hypertension). No new diagnoses of DM1 were recorded. One patient was diagnosed with possible Crohn's disease after FMT. Two patients cleared MDRO after FMT; no clearance in the RBT group	Up to 5 years	Survival: aHR = 1.03; 95% CI: 0.68-1.56. Hospital admission: aHR = 0.92; 95% CI: 0.72-1.18. Cancer: HR = 1.82; 95% CI 0.54-6.09. Diabetes mellitus type 2: HR = 3.44; 95% CI: 0.38-31.02. MDRO: HR = 1.05; 95% CI: 0.20-5.45	Non-randomized, retrospective study design with potential selection bias. Lack of data on clinical resolution following treatment. Retrospective design may miss diagnoses or treatments in primary care settings. Lower FMT dose compared to other studies. Most FMT treatments were delivered via enemas, potentially affecting outcomes. Use of different stool banks and administration routes could mask specific adverse effects
Khanna et al. (2022) [19]	Intervention: RBX2660, a live biotherapeutic product. Type of FMT: rectal administration of a broad consortium of live microbes prepared from human stool	Placebo (normal saline)	Treatment success rate: RBX2660 = 70.6%, placebo = 57.5%. Treatment effect: 13.1 percentage point difference. Posterior probability of superiority: 0.991	Sustained clinical response: approximately 90% of participants with treatment success at 8 weeks remained free of CDI recurrence through 6 months in both groups	6 months	Incidence of treatment-emergent AEs: 55.6% (RBX2660), 44.8% (placebo). Most common side effects: abdominal pain, diarrhea. Serious adverse events: 9 participants experienced SAEs, none related to treatment or administration. Deaths: 2, not related to treatment or administration. Major complications: 2 participants experienced major complications after blinded RBX2660 treatment	Use of PCR assay in >70% of patients, potentially leading to false positives and affecting treatment response rates. Limited generalizability due to a small number of non-White participants and the exclusion of people with irritable bowel syndrome, inflammatory bowel illness, and immunocompromised individuals. Higher placebo response rate, possibly due to enrollment of participants after only one CDI recurrence, indicating less severe dysbiosis
Dubberke et al. (2018) [20]	Intervention: RBX2660, a standardized microbiota-based drug. Type of FMT: enema-based administration. Administration: 2 doses of RBX2660 (Group A), 2 doses of placebo (Group B), or 1 dose of RBX2660 followed by 1 dose of placebo (Group C). Timing: first dose 24-48 hours after CDI treatment antibiotics, second dose 7 ± 2 days later	The control/comparison group B received 2 doses of placebo. The placebo consisted of normal saline and formulation solution in the same proportions found in RBX2660	Prevention of rCDI for 8 weeks following treatment. Efficacy rates: Group A = 61%, Group B = 45%, Group C = 67%. P-value for primary endpoint: p = 0.152. P-value for Group C vs. Group B: p = 0.048. Overall efficacy for RBX2660-treated participants: 88.8%	Efficacy of group C compared to group B: p = 0.048. Combined efficacy in blinded and open-label phases: 88.8%. Efficacy of at least one dose of RBX2660 compared to placebo: p = 0.047. No additional benefit from a second dose within 7 days: p > 0.59	24 months	Total AEs during blinded treatment phase: 379. Percentage of participants with AEs: 64.1%. Most common AEs: gastrointestinal disorders. Safety follow-up duration: mean of 8.3 months. Overall safety profile: favorable, with 90.8% of AEs being mild to moderate	The primary goal was not reached since there was no statistically significant difference between the treatment and placebo groups. Higher-than-expected response rate in the placebo group reduced the study's power to demonstrate efficacy. Heterogeneity in CDI laboratory diagnostic practices may have affected recurrence identification. Lack of positive laboratory diagnosis for some treatment failures could have biased results towards the null. Reliance on NAAT for diagnosis may have led to overdiagnosis
Hvas et al. (2019) [22]	FMT following 4–10 days of taking 125 mg of vancomycin four times a day. Type of FMT: frozen-thawed single-donor solution of donor feces (50 g). Delivery methods: colonoscopy or nasojejunal tube	Fidaxomicin and vancomycin are the control/comparison groups in this study	Combined clinical resolution and negative CD toxin test result at 8 weeks. FMTv: 71% (17/24). Fidaxomicin: 33% (8/24). Vancomycin: 19% (3/16). P-values: FMTv vs. Fidaxomicin: p = 0.009; FMTv vs. vancomycin: p = 0.001; fidaxomicin vs. vancomycin: p = 0.31	Clinical resolution at week 8: 22 (92%, 95% CI: 73-99) for FMTv, 10 (42%, 95% CI: 22-63) for fidaxomicin, 3 (19%, 95% CI: 4-46) for vancomycin; p = 0.0002 for FMTv vs. fidaxomicin, p < 0.0001 for FMTv vs. vancomycin, p = 0.13 for fidaxomicin vs. vancomycin. Negative CD test at week 8: 17 (71%, 95% CI: 49-87) for FMTv, 11 (46%, 95% CI: 26-67) for fidaxomicin, 5 (31%, 95% CI: 11-59) for vancomycin; p = 0.08 for FMTv vs. fidaxomicin, p = 0.01 for FMTv vs. vancomycin, p = 0.36 for fidaxomicin vs. vancomycin. Resolution of diarrhea or diarrhea with negative CD test at week 8: 22 (92%, 95% CI: 73-99) for FMTv, 13 (54%)	8 weeks	Immediate complications of FMTv: 14 (58%) had no side effects; 10 (42%) experienced transient abdominal pain, bloating, constipation, or diarrhea. One SAE related to FMTv: a 50-year-old woman developed a sepsis-like condition. During the 8-week follow-up, 29 (45%) of all patients experienced a total of 48 AEs. No deaths occurred. No AEs specifically related to antibiotic treatments	Absence of patients infected with CD ribotype 027, which may limit generalizability to populations with a high frequency of this ribotype. Study interventions were unblinded, potentially leading to observer bias in reporting
Feuerstadt et al.(2024) [21]	Intervention type: oral microbiome therapeutic composed of purified Firmicutes spores (SER-109). Dose: approximately 3×10^7 ^spore colony-forming units administered as four oral capsules once daily over 3 consecutive days	Placebo	The primary outcome was that SER-109 was superior to placebo in reducing the risk of CDI recurrence up to 8 weeks after treatment, with a recurrence rate of 12% in the SER-109 group versus 40% in the placebo group (relative risk, 0.32; 95% CI, 0.18 to 0.58; p < 0.001).	Sustained clinical response: a higher percentage in the SER-109 group (88%) compared to the placebo group (60%). Engraftment of spore-forming Firmicutes bacteria was observed as early as week 1 and persisted through week 8. Changes in bile-acid profiles: greater increases in secondary bile acids in the SER-109 group	8 weeks	Most adverse events were mild to moderate and gastrointestinal in nature. No serious adverse events were related to SER-109. Adverse events occurred in slightly more than half of the patients in each group. Three deaths in the SER-109 group were not deemed drug-related. Safety profile similar to placebo	Low representation of minority populations in the study. Absence of a stool specimen before antibiotic treatment limits understanding of SER-109's effect on the pre-antibiotic microbiome
Kelly et al. (2016) [18]	Type of FMT: heterologous (donor stool) and autologous (patient's stool). Method of administration: colonoscopy. Dose: mean of 64 g (range 20-100 g) for the donor. FMT procedure: fecal suspension administered into the terminal ileum or cecum	Autologous FMT (using the patient's stool) served as the control/comparison group in this study	Clinical cure rate - donor FMT: 90.9% (95% CI: 69.2% to 97.8%); autologous FMT: 62.5% (95% CI: 41.6% to 79.6%) p-value: 0.042	Chills were reported more frequently after autologous than donor FMT (p = 0.053). Rates of other solicited AEs did not differ significantly between groups. No SAEs were directly related to FMT. Five patients reported new medical conditions or changes in established conditions at the 6-month follow-up, none related to FMT	8 weeks	No SAEs related to FMT. Chills were more frequently reported after autologous FMT (p = 0.053). No significant difference in other solicited AEs between groups. Four SAEs were reported, none related to FMT or colonoscopy. Five patients reported new medical conditions or changes, none related to FMT	The study included only patients with 3 or more recurrences and excluded those aged 75 years or older and immunocompromised individuals. No data were collected on the severity of prior CDI episodes or baseline antibody titers. The study did not meet its target enrollment number. The doses of stool administered varied. PCR testing may lead to overdiagnosis and overtreatment. The study was not powered to detect rare safety outcomes
Cammrota et al. 2015 [5]	FMT via colonoscopy preceded by a short regimen of vancomycin (125 mg four times a day for 3 days). Type of FMT: fecal infusions via colonoscopy	The control/comparison group in this study is the group receiving the standard vancomycin regimen	Primary outcome: resolution of diarrhea related to CDI 10 weeks after treatment; FMT remission rate: 90%; vancomycin remission rate: 26%; OR: 25.2, CI: 1.26 to 502.30, p-value: p < 0.0001	Toxin negative without rCDI at 5 and 10 weeks. FMT: 18/20 patients had negative stool toxins at both 5 and 10 weeks. Vancomycin: 3/19 patients were negative at 5 weeks, 5/19 at 10 weeks; OR: 25.2 (99.9% CI: 1.26 to 502.30); P < 0.0001	10 weeks	No significant adverse events were observed in either group. Temporary symptoms like diarrhea, bloating, and abdominal cramping occurred in 94% and 60% of patients, respectively, but resolved within 12 hours	Lack of blinding for participants and investigators. Vancomycin patients did not undergo colonoscopy, and the presence of PMC in this group is unknown

**Figure 3:**
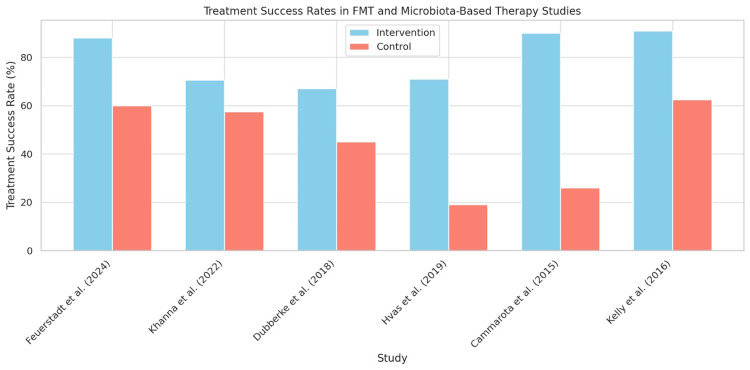
Effectiveness of microbiota-based interventions in preventing recurrent CDI Treatment success rates from six randomized controlled trials comparing FMT or microbiota-based therapies (blue bars) with standard treatments (red bars). Success was defined as the resolution of symptoms without recurrence during the follow-up period CDI: *Clostridioides difficile* infection; FMT: fecal microbiota transplantation

The comparative analysis of six RCTs evaluating microbiota-based therapies for the prevention of rCDI demonstrated consistently higher treatment success rates in intervention groups compared to controls. Reported success rates ranged from 70% to 91% among those receiving interventions such as FMT, SER-109, or RBX2660, whereas control groups exhibited notably lower success rates, ranging from 23% to 62%. These findings underscore the effectiveness of microbiota restoration strategies in reducing recurrence rates of CDI (Figure 3).

The safety profile of microbiota-based therapies for rCDI appears generally favorable, with most adverse events being mild and self-limiting. Gastrointestinal symptoms-such as diarrhea, abdominal pain, and flatulence-were the most frequently reported across interventions, particularly with FMT and microbial consortia products like CP101 and SER-109. Serious adverse events (SAEs) were rare and typically deemed unrelated to the interventions. Notably, several studies reported no adverse events, underscoring the tolerability of these therapies. Overall, the incidence and nature of side effects suggest that microbiota-based interventions are safe, with minimal risk of serious complications.

Discussion

This systematic review provides a comprehensive synthesis of current evidence concerning the safety and efficacy of FMT and related microbiota-based therapies in managing rCDI. The findings highlight key differences in study designs, patient populations, and intervention protocols, while consistently supporting the therapeutic potential of microbiota restoration in rCDI management.

Comparative Analysis of Study Designs and Methodological Approaches

The included studies varied in design, with RCTs primarily assessing short-term efficacy (8-10 weeks), whereas the cohort study provided long-term safety data over five years [17]. The RCTs employed different control groups-placebo [19,21], standard antibiotics [5,22], and autologous FMT [18], which influenced the magnitude of treatment effects observed. Notably, studies using placebo controls demonstrated more conservative efficacy estimates (e.g., 70.6% success with RBX2660 vs. 57.5% placebo) compared to those using antibiotic comparators (90% FMT vs. 26% vancomycin). This suggests that the choice of comparator significantly impacts perceived treatment benefits.

The cohort study by Cold et al. (2022) offered unique insights by comparing FMT to rectal bacteriotherapy (RBT), a non-FMT microbial intervention [17]. The lack of significant differences in long-term outcomes (mortality, hospitalizations, new disease onset) between FMT and RBT suggests that both approaches may have comparable safety profiles, though efficacy was not directly assessed. This contrasts with RCTs, which focus on short-term resolution of rCDI, highlighting a gap in long-term efficacy data.

Heterogeneity in FMT Preparation and Administration

The included studies exhibited considerable variation in FMT protocols, particularly regarding donor material, administration routes, and product standardization. While some trials utilized fresh donor stool [18], others employed frozen preparations [17,22], reflecting ongoing debate regarding optimal stool processing methods. Frozen FMT has gained traction due to logistical advantages, including ease of storage and broader donor availability, yet concerns persist about potential microbial viability loss compared to fresh samples.

Administration routes also differed significantly across studies, with interventions delivered via colonoscopy [5], nasojejunal tube [22], enema [17], and oral capsules [21]. Invasive methods, such as colonoscopy, may enhance proximal colonic engraftment but pose higher procedural risks, whereas oral capsules offer non-invasive convenience but raise questions about gastric acid resistance and distal colonization efficacy. Notably, newer FDA-approved microbiota-based therapies, such as SER-109 (purified Firmicutes spores) and RBX2660 (a defined microbial consortium), represent a shift toward standardized, pharmaceutical-grade formulations, reducing variability inherent in traditional donor-derived FMT.

Despite these methodological disparities, treatment efficacy remained consistently high across studies, suggesting that microbial ecosystem restoration-rather than specific delivery mechanisms-drives therapeutic success.

Heterogeneity in Study Design and Its Impact on Findings

The included studies exhibited considerable variation in protocols, which complicates direct comparisons but also provides valuable insights into the robustness of microbiota-based therapies. A critical appraisal of this heterogeneity is essential to interpret the overall efficacy estimates. We narratively explored outcomes based on three key sources of variation: the choice of comparator, the administration route, and the type of microbial product.

Impact of comparator choice: The choice of control group significantly influenced the magnitude of the observed treatment effect. Studies comparing FMT to standard antibiotics showed the most dramatic differences in efficacy (e.g., 90% remission with FMT vs. 26% with vancomycin in Cammarota et al. [5]). In contrast, trials using a placebo control reported more modest, albeit still clinically significant, treatment effects (e.g., a 13.1 percentage point difference for RBX2660 in Khanna et al. [19]). The use of autologous FMT as a comparator in the Kelly et al. [18] study (90.9% donor vs. 62.5% autologous) effectively isolates the therapeutic benefit of a healthy donor microbiome from the procedural effects of transplantation itself. This variation underscores that while FMT is consistently superior, the reported effect size is highly dependent on the comparator's baseline efficacy.

Influence of administration route: Interventions were delivered via multiple routes, including colonoscopy, enema, nasojejunal tube, and oral capsules. Despite the differences in invasiveness and potential for microbial delivery to different gut regions, efficacy remained high across methods. For instance, the oral capsule SER-109 (Feuerstadt et al. [21]) achieved a sustained clinical response of 88%, which is comparable to the 90-91% cure rates seen in studies using more invasive colonoscopy-based delivery (Cammarota et al. [5], Kelly et al. [18]). This suggests that the core mechanism of microbial ecosystem restoration may be more critical than the specific delivery method, although patient preference and procedural risk remain important considerations.

Traditional FMT vs. standardized microbiome therapeutics: Our review included both traditional donor-derived FMT and newer, standardized, FDA-approved products like SER-109 and RBX2660. While both approaches were effective, the standardized products offer a crucial advantage by reducing the variability inherent in donor stool. However, the reported efficacy for RBX2660 (70.6% in Khanna et al. [19]) was lower than the ~90% rates often cited for traditional FMT. This could be due to differences in the microbial consortium, patient population, or the high placebo response rate in that trial. This highlights a key area for future research: directly comparing standardized products against traditional FMT in head-to-head trials.

Comparison With Other Evidence

Our systematic review evaluates FMT alongside FDA-approved, novel microbiota-based therapies such as SER-109 and RBX2660, representing a significant advancement in the treatment landscape of recurrent rCDI. While earlier reviews by Iqbal et al. (2018) [23], Gupta et al. (2022) [24], and Du et al. (2021) [25] focused primarily on the short-term safety and effectiveness of FMT delivered through various administration routes. They did not address encapsulated delivery methods or emerging live biotherapeutic products. In contrast, our review encompasses both short- and long-term outcomes, with follow-up extending up to five years.

The efficacy reported in previous systematic reviews, approximately 85-90% in Iqbal et al. (2018) [23] and Gupta et al. (2022) [24], 85% in Du et al. (2021) [25], and 90% in Kassam et al. (2013) [26], with the latter also highlighting a substantial difference in favor of FMT over antibiotics (~30%), is consistent with our findings. In our analysis, the clinical cure rate with donor FMT was 90.9%, as reported in Kelly et al. (2016) [18], while Cammarota et al. (2015) [5] demonstrated an FMT remission rate of 90% compared to 26% with vancomycin. Notably, the treatment success rate for RBX2660 was lower at 70.6%. Foundational studies such as Kassam et al. [26] were based predominantly on non-randomized designs. While they did not assess novel live biotherapeutic products, capsule-based FMT has since been evaluated in randomized trials and systematic reviews, demonstrating comparable efficacy to other delivery methods.

Clinical Implications of the Findings

The findings of this systematic review underscore the efficacy of FMT and microbiota-based therapies in preventing rCDI, with treatment success rates ranging from 70% to 91% compared to 23%-62% for standard therapies. These findings lend credence to the use of FMT as a second-line treatment for rCDI in clinical settings, especially for patients who have recurrent recurrences or who are resistant to traditional antibiotics. The safety profile of FMT and investigational microbiome therapeutics (e.g., SER-109, RBX2660) was favorable, with predominantly mild gastrointestinal adverse events and rare serious complications, reinforcing their suitability for widespread use. Given the superior efficacy of donor FMT over autologous transplantation (90.9% vs. 62.5% cure rates), standardized donor screening and stool preparation protocols should be prioritized to ensure consistent therapeutic outcomes. Additionally, the lack of long-term adverse effects in the five-year follow-up by Cold et al. (2022) [17] alleviates concerns regarding potential risks such as infections or metabolic sequelae, further supporting FMT as a durable solution for rCDI. Clinicians should consider early referral for FMT in high-risk populations, including elderly patients and those with comorbidities, to reduce hospitalizations and healthcare costs associated with prolonged antibiotic use.

Future Research Directions

Despite these advances, critical knowledge gaps remain that warrant further investigation. First, RCTs comparing different FMT administration routes (e.g., oral capsules vs. colonoscopy) are needed to optimize delivery methods for efficacy, patient adherence, and cost-effectiveness. Additionally, the lack of standardized protocols complicates direct cross-study comparisons and underscores the need for consensus on optimal preparation, dosing, and administration in future trials. Addressing these heterogeneities will be critical for refining clinical guidelines and ensuring reproducible outcomes in real-world practice.

Second, mechanistic studies should explore the specific microbial taxa and metabolic pathways (e.g., bile acid modulation, short-chain fatty acid production) responsible for FMT’s therapeutic effects, which could inform the development of targeted microbiome therapeutics. Third, long-term (>5-year) safety studies are essential to monitor rare but serious outcomes, such as autoimmune or metabolic disorders, particularly in immunocompromised populations currently underrepresented in trials. Fourth, standardized diagnostic criteria for rCDI (e.g., harmonizing PCR vs. toxin testing) must be established to reduce heterogeneity in clinical trial endpoints. Finally, comparative effectiveness research should evaluate FMT against emerging therapies, such as monoclonal antibodies (e.g., bezlotoxumab) or live biotherapeutic products, to refine treatment algorithms. Addressing these priorities will enhance the precision, accessibility, and safety of microbiome-based interventions for rCDI and other dysbiosis-related conditions.

*Strengths and Limitations of Included Studies* 

The included studies demonstrated several methodological strengths that enhance the validity of their findings. All six RCTs employed rigorous randomization procedures, with three achieving low risk of bias across all Cochrane RoB 2 domains, ensuring robust internal validity. The large sample sizes (e.g., n = 289 in Khanna et al. [19]) and multicenter designs (e.g., U.S./Canada collaborations in Feuerstadt et al. [21]) improved generalizability, while standardized efficacy endpoints (e.g., eight-week rCDI recurrence) facilitated cross-study comparisons. The retrospective cohort study by Cold et al. [17] further strengthened the evidence by providing long-term (five-year) safety data, addressing a critical gap in RCTs. However, limitations were notable: (1) diagnostic heterogeneity, with variable use of PCR (risk of false positives) versus toxin assays for CDI confirmation; (2) residual confounding in observational designs (e.g., unmeasured antibiotic exposures in Cold et al. [17]); and (3) selection bias due to exclusion of immunocompromised patients in most RCTs, limiting applicability to high-risk populations. Additionally, three RCTs had "some concerns" in RoB 2 assessments, primarily due to deviations from intended interventions (e.g., protocol violations in Dubberke et al. [20]).

This review adhered rigorously to PRISMA 2020 guidelines [12], employing a comprehensive search across seven databases and independent dual-reviewer screening to minimize selection bias. The inclusion of both RCTs and a large cohort study provided a balanced evaluation of efficacy and long-term safety, while narrative synthesis accounted for clinical and methodological heterogeneity. However, limitations include the use of a “Full text” filter and restriction to English-language publications, which may have excluded relevant studies published in other languages or those without freely accessible full texts. Additionally, the absence of Embase from our search strategy is a key limitation, which may have resulted in missing relevant studies, particularly those not indexed in PubMed or other databases searched. The inability to conduct meta-analysis due to heterogeneity in interventions (e.g., FMT methods, microbial consortia) and outcomes further constrained quantitative synthesis. Despite these limitations, the review’s focus on contemporary evidence from 2015-2025 and explicit risk-of-bias assessments strengthens its contribution to guiding clinical practice and future research. Future reviews should consider including Embase to enhance the comprehensiveness of the literature search.

## Conclusions

This systematic review confirms that both traditional FMT and novel standardized microbiome therapies are highly effective interventions for rCDI, yielding clinical success rates (70-91%) that are markedly superior to those of antibiotics or placebo (23-62%). The outperformance of donor over autologous FMT further emphasizes that therapeutic success is driven by the restoration of a healthy microbial ecosystem. Nevertheless, a critical finding of this review is the substantial methodological heterogeneity across studies. Variations in comparators, administration routes, and formulations preclude the calculation of a single pooled efficacy estimate and demand a nuanced interpretation of the evidence. Despite these variations, safety profiles were consistently favorable, characterized by mild gastrointestinal adverse events and an absence of significant long-term risks. Clinically, these findings support the integration of microbiota-based therapies as a key second-line treatment for rCDI, particularly for patients with refractory or multiple recurrences. To further advance their role in practice, future research must address several critical gaps. Priorities should include (1) standardizing FMT protocols and conducting head-to-head trials of different delivery methods; (2) performing long-term mechanistic studies to understand microbial dynamics post-transplantation; and (3) expanding clinical trials to include high-risk, immunocompromised populations. Ultimately, advancing these research frontiers will be essential to fully realize the potential of FMT as a transformative, curative, and cost-effective strategy that can disrupt the cycle of antibiotic dependency in rCDI management.
